# Effects of progressive *versus* consistent dose of caffeine ingestion on volleyball players’ exercise performance adaptations following plyometric jump training

**DOI:** 10.3389/fnut.2025.1629950

**Published:** 2025-07-29

**Authors:** Ganfang Zhu, Keqi Fu, Yangzheng Xie

**Affiliations:** Zhejiang Chinese Medical University, Hangzhou, Zhejiang, China

**Keywords:** stretch-shortening cycle, supplementation, physical performance, volleyball players, plyometric jump training

## Abstract

**Background:**

The consumption of caffeine (CAF) to enhance adaptations in athletes has been documented in prior studies; however, the identification of an optimal approach for CAF supplementation during short-term interventions remains unclear. This study aimed to investigate how different caffeine (CAF) dosage strategies consumed 1 h before plyometric jump training (PJT) affect exercise performance adaptations in male volleyball athletes.

**Methods:**

A total of thirty-two young volleyball players volunteered for the study and were randomly divided into four groups: 3 mg/kg of caffeine (CAF-3, *n* = 8), 6 mg/kg of caffeine (CAF-6, *n* = 8), progressively increases from 3 to 6 mg/kg of caffeine (CAF-3 to 6, *n* = 8), and placebo (PL, *n* = 8). All athletes engaged in a 4-week PJT intervention, three times a week, and ingested their CAF or PL in a double-blind manner 1 hour prior to the PJT sessions. Countermovement vertical jump (CMVJ), spike jump (SPJ), block jump (BLJ), 10-m sprint, *T*-test change of direction speed (T-CODS), maximal strength, and Wingate anaerobic power test were assessed both before and after the training intervention.

**Results:**

The CAF-3 to 6 and CAF-6 groups showed more gains (*p* < 0.05) than the CAF-3 and PL groups in the CMVJ (7.6 and 6.9% vs. 4.3 and 4.1%), SPJ (2.5 and 2.3% vs. 1.4 and 1.4%), BLJ (1.1 and 1.0% vs. 0.7 and 0.7%), 10-m sprint (−7.5% and −7.4% vs. −5.2% and −5.0%), T-CODS (−5.4% and −5.2% vs. −3.8% and −3.5%), 1RM strength (5.2 and 5.3% vs. 2.5 and 2.4%), peak power (10.1 and 9.4% vs. 6.2 and 6.0%) and mean power (8.7 and 8.2% vs. 5.1 and 4.9%), respectively, following the training period. However, no significant (*p* > 0.05) differences were observed between the CAF-3 to 6 *versus* CAF-6 in the magnitude of adaptations in the performance of players after training period.

**Conclusion:**

In summary, elevated caffeine intake serves as an ergogenic aid to enhance exercise performance adaptations in volleyball athletes. Furthermore, a progressive CAF loading strategy appears to be as effective as maintaining a consistent dosage throughout a 4-week PJT program. This approach presents a viable option for CAF supplementation, potentially attenuating total CAF usage while eliciting similar training benefits.

## 1 Introduction

Volleyball is defined as a high-intensity, intermittent sport that requires lower body muscular strength and power, along with jump ability and sprint performance over short distances (1). Plyometric jump training (PJT) is an effective training strategy that can improve muscular strength and power, jumping ability, sprinting speed, and change of direction ability in volleyball players (2, 3). In addition to critical variables such as training intensity, volume-load, frequency, training surface, and other program design considerations for designing an optimal PJT program (4–7), some strength and conditioning coaches in volleyball also aim to incorporate sports supplementation to achieve further improvements in physical performance during the short-term preparation phase (8). Indeed, when training time is limited, sports supplementation may be recommended to facilitate performance improvements (9).

While knowledge regarding the effects of sports supplements on athletic performance is continually expanding, it is well-established that caffeine (CAF) significantly contributes to enhancing sports performance and improving physical capabilities related to sports by promoting neuromuscular (i.e., increase the contractile properties of the muscle fibers) and metabolic (i.e., improvements in Na^+^–K^+^ pump) adaptations (10–12). In fact, research indicates that CAF supplementation is effective in enhancing adaptations related to jumping ability, sprinting, change of direction, and maximal strength in volleyball players who undergo various training programs (13–15).

Prior studies have explored both the immediate and long-term effects of CAF on athletes’ physical performance, employing different doses based on body mass (BM), which has resulted in inconsistent conclusions (10–15). For example, a dosage of 3 mg per kg of BM has been identified as optimal (16), while male athletes showed significant performance improvements with a dosage of 6 mg per kg of BM (10). Research by Nemati et al. (14) indicated that a 6 mg per kg of BM intake led to better performance in volleyball-specific assessments compared to the 3 mg per kg of BM dosage. Furthermore, Wu and Jiang (17) reported that basketball players who consumed 6 mg per kg of BM before PJT exhibited greater physical performance adaptations than those who ingested 3 mg per kg of BM.

Although previous research has generally examined CAF dosages ranging from 3 to 6 mg per kg of BM (10–17), the effects of gradually increasing CAF doses on exercise performance adaptations following a 4-week PJT program in volleyball players remain uncertain. In fact, progressively increasing the dosage from 3 to 6 mg per kg of BM over a 4-week period may serve as an effective strategy, emphasizing the body’s improved tolerance to CAF absorption throughout the training phase (10, 18, 19). This approach could be more beneficial than maintaining a high and constant dose through a duration, which may lead to gastrointestinal issues, feelings of anxiety, or other side effects (10, 12). Indeed, caffeine’s ergogenic effects are thought to be mediated through various mechanisms, including modulation of the Na^+^/K^+^ pump and antagonism of adenosine receptors, leading to increased neuronal excitability and reduced perception of fatigue (10–14). The current study aimed to explore a progressive CAF intake approach, hypothesizing that a gradual increase in dosage may allow for a more controlled adaptation of these systems. Specifically, it is proposed that this method could enhance tolerance by allowing adenosine receptors to gradually downregulate in response to increasing CAF levels, or reduce side effects by minimizing the initial shock to the nervous system associated with a sudden high dose (12, 17). While the performance-enhancing effects of CAF are well-established (13–16), the optimal method of administration remains a subject of investigation. This study focuses on a progressive CAF intake strategy, driven by the hypothesis that a gradual increase in dosage may improve tolerance and reduce side effects compared to a constant dosage. This may be attributed to several mechanisms, including a potential for gradual desensitization of adenosine receptors, which are responsible for caffeine’s stimulatory effects, or a more controlled physiological adaptation to the stimulant, minimizing the likelihood of adverse reactions (15, 17, 18). It is important to note that these assumptions are hypothetical and require investigation. Therefore, this study sought to investigate various strategies for CAF supplementation, specifically comparing a consistent dosage of 3 mg and 6 mg per kg of BM with a progressive increase from 3 mg to 6 mg per kg of BM over the course of 4 weeks during the preparations phase of training using the PJT intervention. We hypothesize that a gradual increase in CAF dosage may serve as an effective approach to enhance exercise performance adaptations in volleyball players during the short-term PJT program.

## 2 Materials and methods

### 2.1 Registration and ethics approval

Participants were given detailed information about the possible risks and discomforts related to the study and also were required to sign informed consent statements. As the study protocol incorporated participants identified as “athletes” receiving supplements to examine its effects on the athletic performance, in accordance with the ethics committee of Zhejiang Chinese Medical University, this study did not enter clinical trials that require registration, and the study was approved by the Ethics and Medical Committees of the University (20240411364AA), as well as conform to the latest version of the Declaration of Helsinki regarding human subjects.

### 2.2 Participants

The sample size was calculated utilizing G*Power software (version 3.1.9, Universität Düsseldorf, Germany), which suggested a total of 24 participants to attain a statistical power of 0.95 and a significance level of 0.05 for the *F* tests, particularly focusing on the repeated measures ANOVA for within-between interactions. This determination was founded on an effect size of 0.25, as noted in a study examining the effects of CAF supplementation and PJT on athletes’ physical performance (17). To address the possibility of participant attrition during data collection, the sample size was later increased to 32 athletes (*n* = 8 for each group). As a result, 32 young male volleyball players with similar fitness levels and training regimens from local academies volunteered for the study and were randomly assigned into four groups: 3 mg/kg of caffeine (CAF-3, *n* = 8), 6 mg/kg of caffeine (CAF-6, *n* = 8), a progressive increase from 3 to 6 mg/kg of caffeine (CAF-3 to 6, *n* = 8), and a placebo group (PL, *n* = 8) (Table 1), using a computer-based random number generator (simple randomization, 1:1:1:1 ratio) which were unpredictable for authors and participants. Each group included 1 setter, 2 middle blockers, 2 outside hitters, and 3 opposite hitters. To be eligible for the study, players had to satisfy specific inclusion and exclusion criteria: (1) they needed to be familiar with PJT but had not engaged in it for 3 months prior to the study’s initiation, (2) they should be free from any medical or orthopedic conditions that could potentially affect their participation or performance, as confirmed by a sports medicine physician (20), and (3) they had to be classified as trained athletes (i.e., Tier 2). Participants were categorized as low caffeine consumers, defined as ingesting less than 50 mg of caffeine daily, consistent with the criteria established by Filip et al. (21). This classification was further validated using a modified version of the questionnaire developed by Bühler et al. (22), which assesses total daily caffeine intake from various dietary sources.

**Table 1:** Descriptive data (mean ± SD) for the experimental groups.

Characteristics	CAF-3	CAF-6	CAF-3 to 6	PL
Age (y)	20.5 ± 1.1	20.6 ± 1.3	20.7 ± 1.2	20.4 ± 1.2
Height (cm)	184.4 ± 3.9	183.7 ± 4.6	185.2 ± 3.3	184.8 ± 4.1
Body mass (kg)	84.2 ± 4.1	82.7 ± 5.6	83.3 ± 4.5	84.7 ± 3.9
Training experience (y)	5.8 ± 0.8	5.7 ± 0.6	6.1 ± 1.1	5.5 ± 0.9

### 2.3 Experimental design

This study utilized a pre/post-test design during the preparation phase of the training plan (i.e., pre-season). Throughout the study period, players engaged in training regimens tailored to their specific playing positions and managed regarding the training loads as PJT included or not. The comprehensive training program, encompassing short warm-up routines, technical and tactical drills, and plyometrics, was meticulously designed and prescribed by the team coaches. All participants executed their respective training sessions concurrently, adhering to the physiological and skill demands inherent to their roles on the field (Table 2). The study duration lasted for 6 weeks including 1 week for familiarization with the study design and procedures, as well as anthropometric measurements (i.e., height with a wall-mounted stadiometer) (Seca 222, Terre Haute, IN, ± 0.5 cm) and body mass with a digital scale (Tanita, BC-418MA, Japan, ± 0.1 kg), 1 week for the pre-test, 4 weeks for the PJT intervention, and 1 week for the post-test. The measurements of exercise performance in players were conducted over 3 days with 48 h intervals between testing sessions as follows: day 1 (i.e., Monday), the measurements of countermovement vertical jump (CMVJ), spike jump (SPJ), block jump (BLJ), and 10-m sprint; day 2 (i.e., Wednesday), evaluations of *T*-test change of direction speed (T-CODS) and the Wingate anaerobic power test; and day 3 (i.e., Friday), assessment of maximal strength performance using 1 repetition maximum (1RM) in the leg press exercise. To ensure adequate recovery, 10 min rest intervals were scheduled between exercise performance tests. Following the pre-test, all athletes participated in a 4-week volleyball training regimen (i.e., 5 days a week), while the PJT was additionally included before regular volleyball practices on Mondays, Wednesdays, and Fridays. Participants ingested their CAF dosages or PL in a double-blind manner 1 hour prior to the PJT sessions. Upon completing the training period, the sequence and order of the pre-intervention tests were repeated, serving as the post-test. Since all tactical and technical volleyball training took place in the afternoon, it was decided that all testing and PJT sessions would also occur in the afternoon to reduce the impact of circadian rhythms on performance. The CMVJ, SPJ, BLJ, 10-m sprint, and T-CODS were performed on a wooden volleyball court, while the 1RM leg press and Wingate test were carried out in a laboratory environment with a temperature of 27 and 29°C and humidity levels of 45 and 55%, all overseen by the same examiner. Participants were required to wear matching footwear and standard training shorts and T-shirts during both the pre and post-tests.

**Table 2:** Training program over the course of the observation period.

Week days	Intervention	Time
Monday	PJT + technical-tactical training	17:00–19:00
Tuesday	Small-sided and simulated competitive games	17:00–19:00
Wednesday	PJT + technical-tactical training	17:00–19:00
Thursday	Recovery	–
Friday	PJT + technical-tactical training	17:00–19:00
Saturday	Small-sided and simulated competitive games	17:00–19:00
Sunday	Recovery	–

### 2.4 CAF supplementation strategies

During the intervention, athletes were administered either caffeine (Nutricost, UT, United States) or polydextrose (Akhil Healthcare Pvt. Ltd., India), depending on their group assignment (CAF or PL, respectively). Both substances were provided in powdered form and meticulously encapsulated in size “00” gelatin capsules. A sensitive scale (Gromy Industry, Co., LTD, China; 0.001 g precision) was utilized to ensure accurate weighing of each capsule’s contents (caffeine or polydextrose). Each capsule weights were calculated based on each participant’s body weight and were filled manually (i.e., manual capsule filling machine [Feton International, Belgium]) by a laboratory member, who remained blinded to the contents. To maintain blinding, all capsules were identical in color and taste. Administration occurred 1 hour prior to the PJT, but not before volleyball training sessions. Participants were instructed to consume the capsules with 100 cc of water. The CAF-3 and CAF-6 groups received CAF dosages of 3 mg/kg and 6 mg/kg of BM, respectively, maintaining a consistent dosage throughout the 4-week training intervention. In contrast, the CAF-3 to 6 group started with 3 mg/kg of caffeine in week 1, increased to 4 mg/kg in week 2, 5 mg/kg in week 3, and finally 6 mg/kg in week 4. Participants were instructed to take their allocated supplements prior to each PJT session, with CAF and PL provided in weekly packets. At the end of each week, all packets had to be returned by the participants, regardless of their usage. It is important to underscore that the capsules were not labeled with any information about their contents, which kept both the researchers and participants blind to the composition until the study was completed (10, 17).

### 2.5 PJT program

All athletes participated in a five-day volleyball training program designed for the preparation phase. Each training session lasted between 100 to 120 min, which included a 15 min warm-up, 75 to 85 min main training activities such as tactical and technical drills, small-sided games, and simulated competitive matches, followed by a 10 min cool-down. The volleyball training session loads were adjusted according to the number of jumps for the training groups and all players regarding their specific positions performed similar jump loads throughout the intervention period. Additionally, the players took part in a PJT program, which consisted of 4 sets of 10 repetitions of squat jumps, depth jumps from a 45-cm box, hurdle jumps, and box jumps from a height of 45 cm (23). The program was implemented three times per week, with 72 h intervals between sessions. This scheduling aligns with previous research interventions that utilized comparable PJT volume loads and intensities, which have been shown to induce physical performance adaptations in athletes (17). To facilitate recovery, a one-minute rest was allotted between sets and a two-minute rest between exercises. Furthermore, to mitigate the potential impact of PJT induced fatigue on the efficacy of volleyball training sessions, the PJT volume load within the training program was carefully adjusted in consideration of the technical and tactical drills and the 10 min rest interval between PJT and volleyball training were assigned to ensure recovery (17). All PJT sessions were conducted on a wooden volleyball court and were closely supervised by a strength and conditioning coach as well as a researcher to ensure adherence to the established training protocols.

### 2.6 Exercise performance measurement procedures

#### 2.6.1 Jumping ability

The measurements for the CMVJ, SPJ, and BLJ were conducted using a wall-mounted vertical jump tester (VERTEC Power System, United States) after a 10 min general warm-up and three sub-maximal jumps for specific warm-ups. The CMVJ test started with participants standing upright. When prepared, they flexed their knees to initiate a downward movement to a depth of their preference (i.e., 90°). They then extended their knees to jump as high as possible. At the peak of their jump, participants made contact with the vanes on the Vertec. The difference between the highest vane contacted during the jump and the participant’s standing reach was calculated (24). In the SPJ, participants were instructed to stand on the floor about 3–4 m from the Vertec device. Upon receiving a command from the examiner, they would initiate their approach run, which could consist of either two or three steps, and execute a complete spiking motion with the palm of their dominant hand (25). The BLJ began from a standing posture, with the hands held at shoulder level and the arms raised without any prior swinging action. When prompted, the players flexed their knees and jumped as high as they could, similar to the BLJs practiced in volleyball. The highest finger contacts on the Vertec for both the SPJ and BLJ were recorded as previously recommended. In each jump performance test, players completed three trials, and the best result from these attempts was recorded for further analysis (26). The reliability coefficient (ICC) for repeated measurements at CMVJ, SPJ, and BLJ were 0.95, 0.93, and 0.94, respectively.

#### 2.6.2 10-m sprint and T-CODS

**Figure 1:**
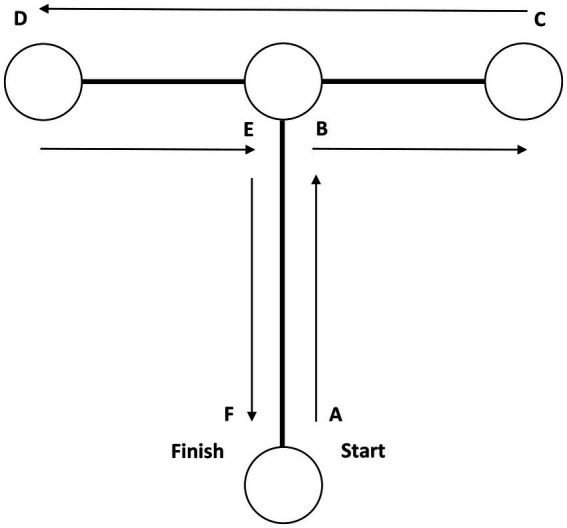
T-CODS procedure.

The time taken for the 10-m linear sprint was measured to the nearest 0.01 s using a single-beam timing gate system (Brower Timing System, United States). Following the 3 trials for warm-ups, the participants commenced from a standing start, positioning the toe of their preferred foot forward and behind the starting line. Timing began when the participant voluntarily initiated the test. The timing gates were placed at the start (0.3 m in front of the starting line) and at the 10-m mark. They were positioned about 0.7 m above the floor (at hip level) to accurately capture trunk movement and avoid false triggers caused by limb movements (27). For the T-CODS, the timing system and procedures were identical to those used for the 10-m sprint, except that participants had to run straight while making several rapid directional changes which identified in the Figure 1 (28). The ICC for repeated measurements at 10-m sprint, and T-CODS were 0.96, and 0.95, respectively.

#### 2.6.3 1RM leg press

The assessment of maximal strength (i.e., 1RM) was conducted using a 45° leg press machine (Nebula Fitness, Inc., Versailles, OH). Each participant began with a preliminary warm-up, performing 5 to 10 repetitions with light resistance. Following this, the resistance was gradually increased, allowing the participant to complete 2 to 3 repetitions. The participant then performed one repetition for each subsequent load increase until reaching the point of voluntary failure. The goal was to achieve a maximal lift within five attempts. A two-minute rest period was provided between each set (29).

#### 2.6.4 Wingate anaerobic power

A cycle ergometer (Ergomedics 874, Monark, Sweden) was employed to conduct the WAnT. After a warm-up lasting 5 min, participants were asked to pedal as quickly as possible for 30 s against a braking force calculated by multiplying their body mass in kilograms by 0.075. The peak power (Ppeak) was assessed by averaging the power over a 5 s period, which generally reflected the highest performance, typically seen in the first 5 s of the test. The mean power (Pmean) was calculated as the average power over the full 30 s interval. Throughout the duration of the test, participants were given verbal motivation to encourage them to perform at their highest capacity for the full duration of 30 s (30).

### 2.7 Control of diet

Participants were required to follow their typical eating patterns during the study. Before any evaluations took place (i.e., in week 1), they were asked to record their food consumption over a span of 3 days. After analyzing the dietary data (Nutritionist IV), participants were instructed to sustain a caloric intake of approximately 3,200 kcal, comprising around 15% protein, 65% carbohydrates, and 20% fat. Additionally, participants were instructed to abstain from any CAF consumption beyond their habitual daily intake, and to avoid all other sports supplements for the duration of the study.

### 2.8 Statistical analysis

The data analysis was performed utilizing SPSS software (Version 24, SPSS Institute, Chicago, IL, United States). The results were expressed as mean ± standard deviation (SD). To assess the normality of the pre- and post-intervention values for the dependent variables, the Shapiro–Wilk Normality test was employed. A 4 (group) × 2 (time) ANOVA was conducted to identify significant differences among the groups regarding the measured variables. In cases where a significant *F* value was found, the Bonferroni *post hoc* test was utilized. Effect sizes (ES) were calculated using Hedges’ g with the 95% confidence interval (CI), and based on the classification proposed by Hopkins et al. (31), an ES of < 0.2 was considered trivial, 0.2–0.6 was small, 0.6–1.2 was moderate, 1.2–2.0 was large, 2.0–4.0 was very large, and > 4.0 was nearly perfect. The percentage changes from pre-training to post-training for each variable were calculated using the formula ((post-intervention – pre-intervention)/pre-intervention × 100) and analyzed through one-way ANOVA and the Bonferroni *post hoc* test. The significance threshold was established at 0.05.

## 3 Results

There were no significant differences among the groups at baseline values (*p* > 0.05). All participants completed the training sessions, and there were no injury reports related to the PJT intervention or testing. Additionally, none of the athletes reported experiencing side effects from CAF supplementation, including nervousness, increased vigor, or gastrointestinal issues.

**Figure 2:**
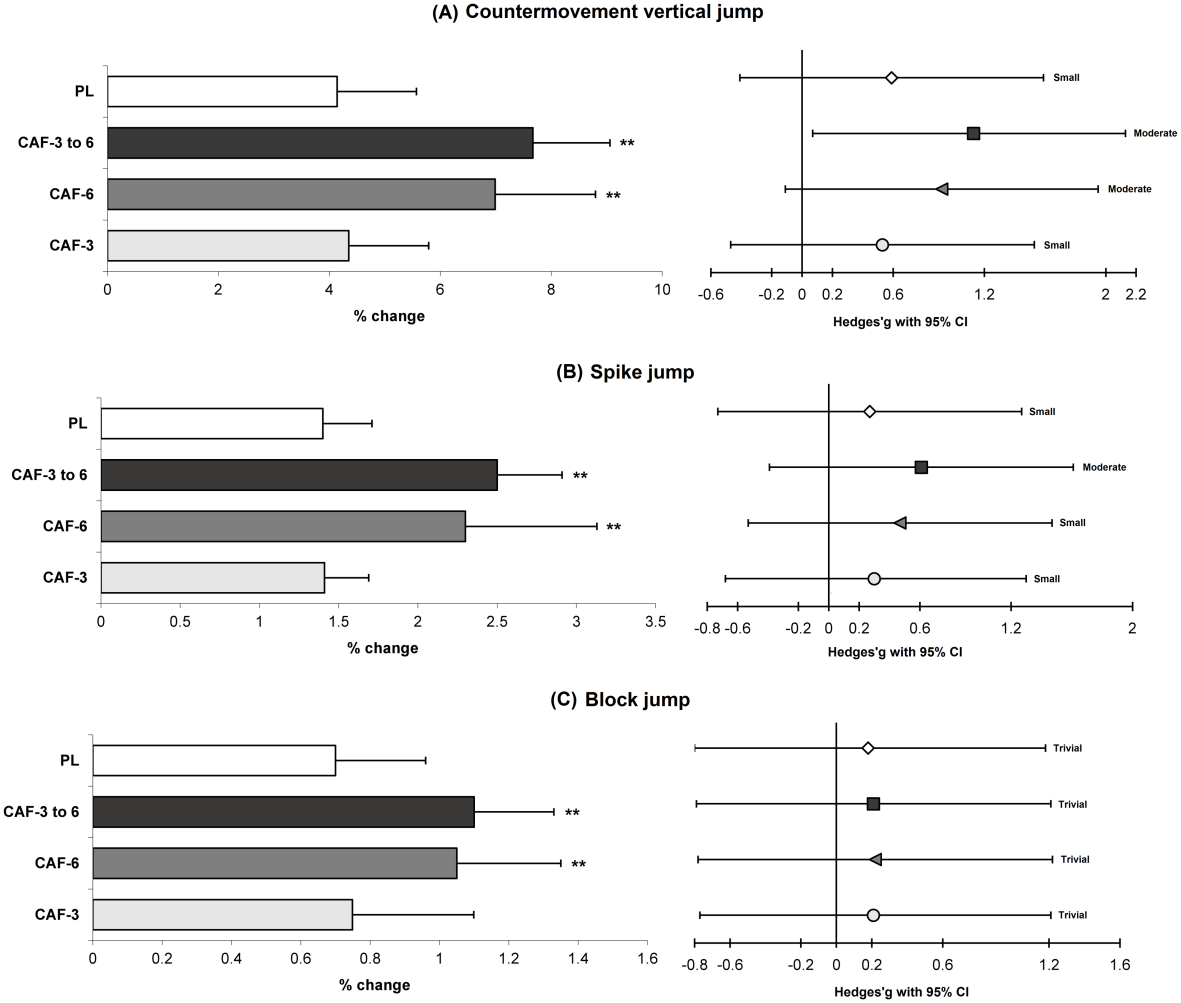
Magnitude of changes (%) and ES with 95% CI in the CMVJ **(A)**, SPJ **(B)** and BLJ **(C)** from pre to post-intervention in the training (mean ± SD). ** Significant differences vs. CAF-3 and PL groups (*p* < 0.05).

**Figure 3:**
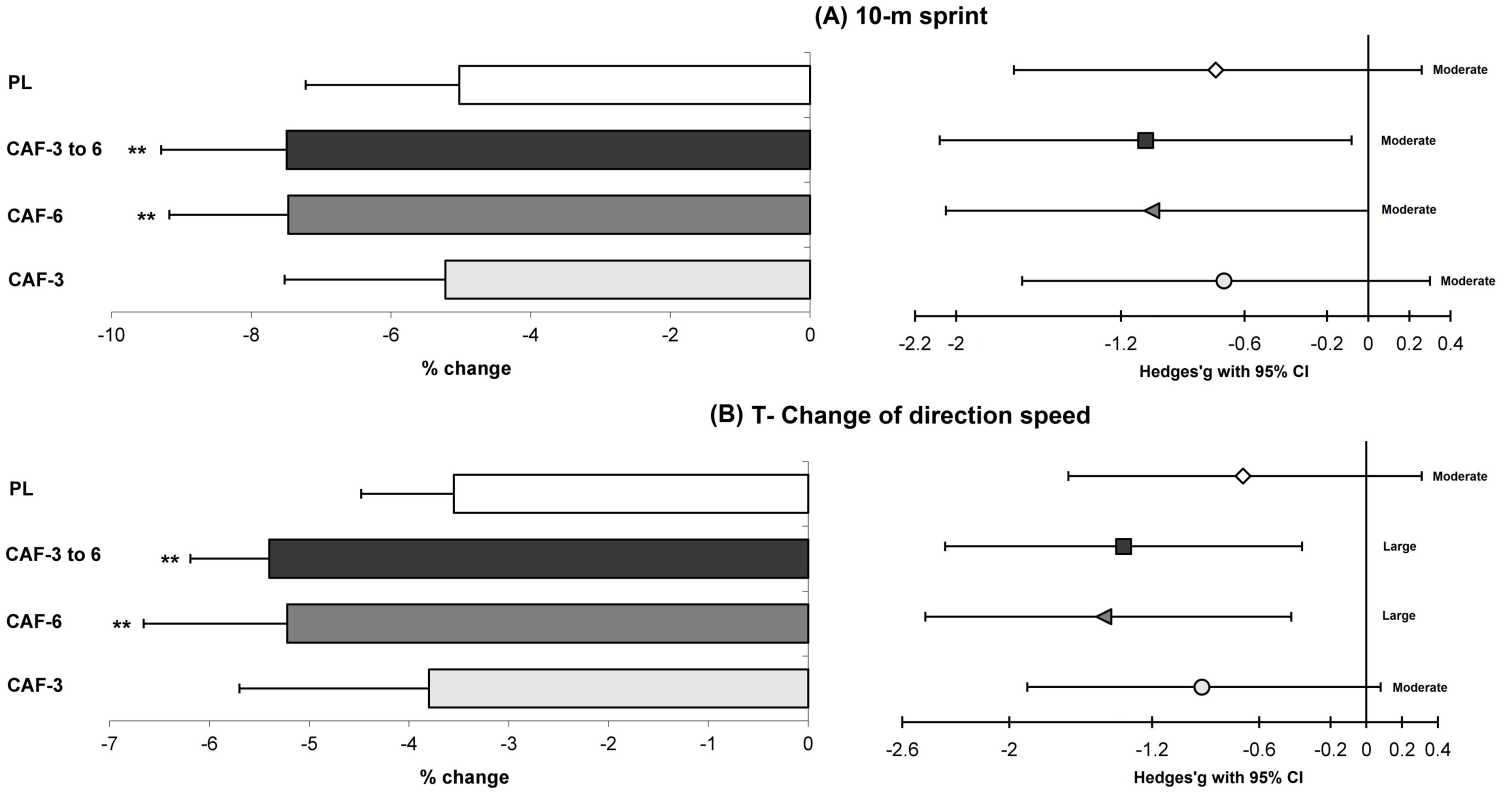
Magnitude of changes (%) and ES with 95% CI in the 10-m sprint **(A)**, and T-CODS **(B)** from pre to post-intervention in the training (mean ± SD). ** Significant differences vs. CAF-3 and PL groups (*p* < 0.05).

**Figure 4:**
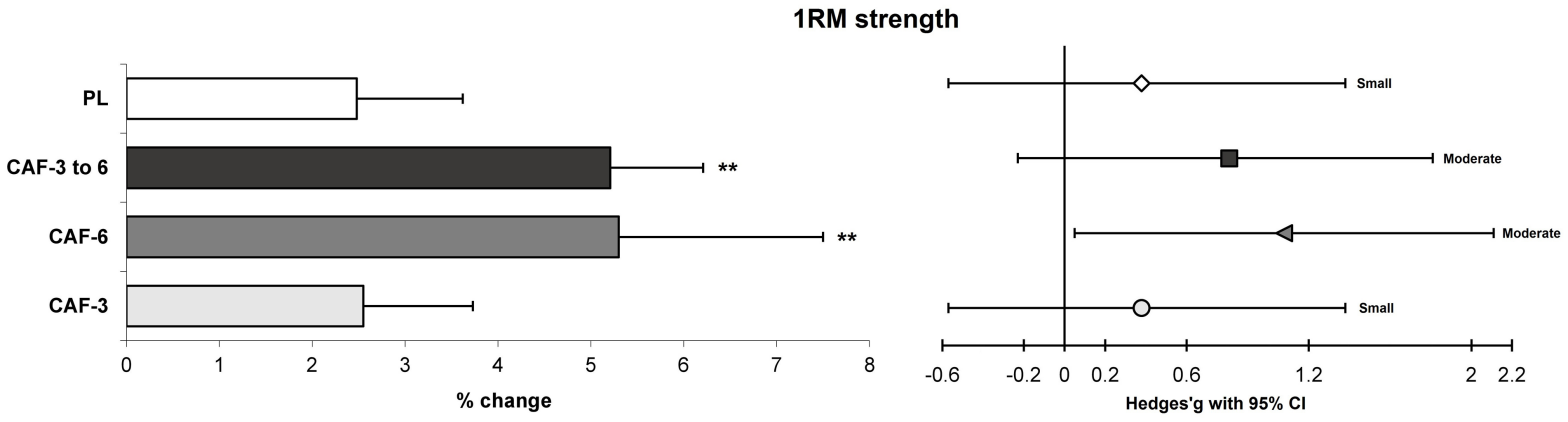
Magnitude of changes (%) and ES with 95% CI in the 1RM strength from pre to post-intervention in the training (mean ± SD). ** Significant differences vs. CAF-3 and PL groups (*p* < 0.05).

**Figure 5:**
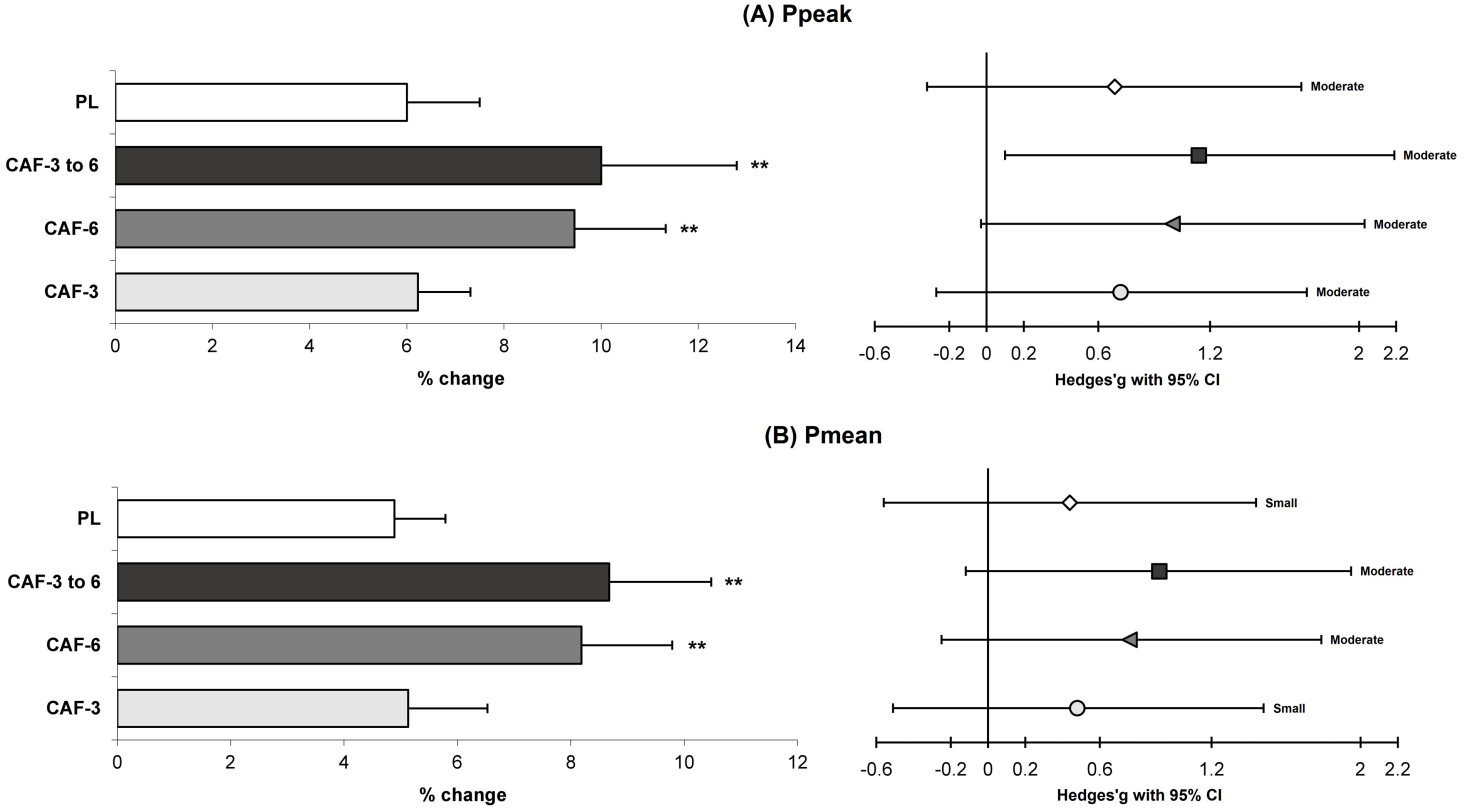
Magnitude of changes (%) and ES with 95% CI in the Ppeak **(A)** and Pmean **(B)** from pre to post-intervention in the training (mean ± SD). ** Significant differences vs. CAF-3 and PL groups (*p* < 0.05).

There was a significant main effect of time (*p* = 0.001) which indicates small to large benefits in exercise performance gains for volleyball players following the 4-week PJT intervention (Table 3; Figures 2–5).

**Table 3:** Changes in exercise performance adaptations from pre to post-training for all training groups (mean ± SD).

Variables	CAF-3	CAF-6	CAF-3 to 6	PL	Significant
CMVJ (cm)					
Pre	45.5 ± 3.3	46.3 ± 3.2	45.8 ± 2.9	45.2 ± 2.9	Group × Time interaction
Post	47.5 ± 3.8*	49.6 ± 3.6*,**	49.3 ± 2.8*,**	47.1 ± 3.1*	*F* = 10.9, *p* = 0.001, n^2^_p_ = 0.540
SPJ (cm)					
Pre	301.1 ± 12.9	301.3 ± 14.6	301.1 ± 11.9	301.5 ± 14.7	Group × Time interaction
Post	305.3 ± 13.2*	308.2 ± 13.3*,**	308.6 ± 11.3*,**	305.7 ± 15.1*	*F* = 11.7, *p* = 0.001, n^2^_p_ = 0.558
BLJ (cm)					
Pre	283.6 ± 9.6	284.2 ± 12.6	283.7 ± 14.1	284.8 ± 10.9	Group × Time interaction
Post	285.7 ± 9.7*	287.2 ± 13.1*,**	286.8 ± 14.2*,**	286.8 ± 10.7*	*F* = 3.84, *p* = 0.020, n^2^_p_ = 0.291
10-m sprint (s)					
Pre	2.72 ± 0.19	2.69 ± 0.17	2.73 ± 0.16	2.71 ± 0.17	Group × Time interaction
Post	2.58 ± 0.19*	2.49 ± 0.19*,**	2.51 ± 0.19*,**	2.58 ± 0.16*	*F* = 3.06, *p* = 0.044, n^2^_p_ = 0.247
T-CODS (s)					
Pre	12.10 ± 0.57	12.08 ± 0.47	12.07 ± 0.48	12.13 ± 0.58	Group × Time interaction
Post	11.62 ± 0.40*	11.45 ± 0.33*,**	11.41 ± 0.44*,**	11.70 ± 0.60*	*F* = 3.14, *p* = 0.041, n^2^_p_ = 0.252
1RM (kg)					
Pre	289.3 ± 17.2	285.0 ± 13.6	288.7 ± 17.4	286.8 ± 16.8	Group × Time interaction
Post	296.8 ± 19.9*	300.1 ± 12.8*,**	303.7 ± 17.6*,**	294.1 ± 19.6*	*F* = 8.80, *p* = 0.001, n^2^_p_ = 0.485
Ppeak (W)					
Pre	812.5 ± 64.3	816.7 ± 69.7	810.2 ± 63.8	813.1 ± 66.6	Group × Time interaction
Post	863.1 ± 68.4*	893.6 ± 75.9*,**	891.2 ± 70.1*,**	861.8 ± 67.3*	*F* = 9.86, *p* = 0.001, n^2^_p_ = 0.514
Pmean (W)					
Pre	473.8 ± 48.0	473.2 ± 48.8	471.5 ± 40.8	475.5 ± 51.0	Group × Time interaction
Post	497.8 ± 46.9*	511.7 ± 46.9*,**	512.2 ± 42.7*,**	498.5 ± 48.7*	*F* = 17.32, *p* = 0.001, n^2^_p_ = 0.650

* Significant differences vs. pre (*p* < 0.05), ** significant differences vs. CAF-3 and PL (*p* < 0.05). CMVJ, countermovement vertical jump; SPJ, spike jump; BLJ, block jump; T-CODS, T-change of direction speed; 1RM, one repetition maximum; Ppeak, peak power output; Pmean, mean power output. PL, placebo; CAF, caffeine.

A significant group by time interaction (*p* = 0.001 to 0.044, n^2^_p_ = 0.247 to 0.650) were observed in between the training groups, which indicates more adaptations for the CAF- 6 and CAF- 3 to 6 groups *versus* CAF-3 and PL groups in the performance adaptations. In fact, the CAF-6 and CAF- 3 to 6 groups showed significantly greater improvements compared with the CAF-3 and PL groups in the CMVJ (7.6% [p = 0.001 and 0.001] and 6.9% [*p* = 0.011 and 0.005] versus, 4.3 and 4.1%) (Figure 2A), SPJ (2.5% [p = 0.001 and 0.001] and 2.3% [*p* = 0.010 and 0.010] versus, 1.4 and 1.4%) (Figure 2B), BLJ (1.1% [*p* = 0.013 and 0.006] and 1.0% [*p* = 0.029 and 0.015] versus, 0.7 and 0.7%) (Figure 2C), 10-m sprint (−7.5% [*p* = 0.023 and 0.015] and −7.4% [*p* = 0.024 and 0.015] versus, −5.2% and −5.0%) (Figure 3A), T-CODS (−5.4% [*p* = 0.019 and 0.007] and −5.2% [*p* = 0.034 and 0.013] versus, −3.8% and −3.5%) (Figure 3B), 1RM strength (5.2% [*p* = 0.008 and 0.006] and 5.3% [*p* = 0.006 and 0.005] versus, 2.5 and 2.4%) (Figure 4), Ppeak (10.1% [*p* = 0.003 and 0.002] and 9.4% [*p* = 0.016 and 0.009] versus, 6.2 and 6.0%) (Figure 5A) and Pmean (8.7% [*p* = 0.001 and 0.001] and 8.2% [p = 0.003 and 0.001] versus, 5.1 and 4.9%) (Figure 5B), respectively, following the training period.

## 4 Discussion

This research aimed to investigate the impact of various CAF supplementation strategies, utilizing different dosages, on the exercise performance adaptations of male volleyball players following a 4-week PJT intervention during the pre-season. The findings revealed that a 4-week PJT intervention could lead to significant improvements in the exercise performance of volleyball players. Furthermore, the greater adaptations were observed when players consumed 6 mg/kg of CAF or when there was a gradual increase in CAF intake from 3 to 6 mg/kg throughout the 4-week intervention. Conversely, the intake of 3 mg/kg of CAF over the 4-week training period did not yield any additional benefits compared to the PL conditions, indicating that this lower dosage does not enhance exercise performance adaptations in volleyball players over a short duration.

Our research corroborates findings from several earlier studies that indicate the efficacy of PJT as a training method for improving athletes’ jumping ability, sprinting speed, and agility, particularly among volleyball players (2, 3, 25, 32). In this investigation, we observed significant small to large ESs in the improvements of CMVJ, SPJ, BLJ, 10-m sprint, and T-CODS following a 4-week PJT intervention for volleyball athletes. These advancements may stem from adaptive changes in neuromuscular characteristics resulting from the PJT program (4–6). Previous studies have suggested that participation in PJT can lead to increased motor unit recruitment, enhanced coordination among various muscle groups, and improved neural activation of the primary muscles involved (5, 33). Furthermore, PJT is linked to the engagement of fast-twitch muscle fibers, increased stride length and frequency, and a swift transition from eccentric to concentric muscle actions, all contributing to small and moderate gains in jumping ability, sprinting speed, and change of direction performance in volleyball players who reported in previous studies (2, 4–6, 25, 33).

Additionally, the ergogenic effects of CAF were only observed in athletes who consumed 6 mg/kg of CAF or those who gradually increased their CAF intake from 3 to 6 mg/kg of over the course of the 4-week intervention. This indicates that a dosage of 3 mg/kg of CAF is insufficient to elicit physiological adaptations associated with caffeine, as it did not yield greater improvements than the PL group. In fact, the adaptations observed in both the PL and CAF-3 groups were similar (i.e., small to moderate ES) throughout the 4-week PJT intervention. Conversely, strategies involving 6 mg/kg of CAF and a gradual increase from 3 to 6 mg/kg of CAF led to superior enhancements (moderate to large ESs) in jumping ability, sprinting, and T-CODS among volleyball players. These results align with previous studies suggesting that for volleyball players or during PJT interventions, a caffeine dosage of 6 mg/kg is more effective than 3 mg/kg in achieving additional gains (13, 14, 17). It appears that ingesting a higher dose of CAF, rather than a lower dose (such as 3 mg/kg of body mass), prior to engaging in PJT has a counterproductive effect on adenosine receptors in both the central and peripheral nervous systems (10). This results in an increased central drive and a reduction in the perception of pain and fatigue during physical activity. Additionally, CAF triggers the release of serotonin in the cerebral cortex, influencing the central nervous system (11). This mechanism enhances the performance of the sympathetic nervous system while inhibiting the activity of certain neurons (12, 14). Consequently, PJT is conducted with increased arousal, leading to enhanced activation of muscle fibers via improvements in Na^+^–K^+^ pump as well as neuromuscular adaptations (14–17) resulting in significant improvements in jump performance, sprinting, and T-CODS.

Our results demonstrated that starting with 3 mg/kg of CAF and increasing to 6 mg/kg yielded similar performance gains as maintaining a constant dose throughout the 4 weeks. These findings may lend support to our assumption regarding the body’s capacity to tolerate and absorb CAF as well as lower CAF ingestions than consistent dose, indicating that athletes may not need to begin their training with high doses of CAF; rather, they could start with a lower dose and progressively increase it each week (18, 19). Nonetheless, it is crucial to recognize that our suggestions are speculative, based solely on a 4-week intervention, and may not be generalized to longer training durations and additional studies are necessary to investigate the effects of different CAF dosage strategies on performance adaptations in other athletes.

The results of the present study showed that a 4-week PJT protocol produced small gains in strength performance, and these gains were the same as those seen in athletes consuming 3 mg/kg of CAF, suggesting that low-dose CAF intake did not provide additional benefits for further strength gains and both the CAF-3 and PL groups showed small ESs following the PJT intervention. In fact, consuming 6 mg/kg of CAF or increasing caffeine from 3 to 6 mg/kg during short-term training was effective in promoting greater gains. In line with previous studies highlighting the positive effects of PJT on strength gains with the same ESs (7, 17), our study suggests that a strategy of using high-dose or gradually increasing CAF doses is optimal for volleyball players in order to achieve greater strength adaptations in short-term interventions (moderate vs. small ES). The greater strength gains after the short-term intervention were mainly attributed to the improvement of the neuromuscular junction (7), as well as the increased recruitment of motor units, the improvement of muscle internal and external coordination, and the enhanced storage and utilization of elastic potential energy (34). In addition, the intake of CAF (i.e., 6 mg/kg or 3 to 6 mg/kg) before PJT may stimulate higher activation of muscle fibers and promote calcium release during exercise (10, 17), thereby enhancing adenosine receptors in the central and peripheral nervous systems, reducing fatigue during training sessions, leading to more muscle fiber recruitment and further improvement in strength performance (12, 17).

More importantly, the intake of CAF, from low to high doses over the course of 4 weeks, produced similar and moderate ES in strength improvements to those seen in athletes who consumed high doses of CAF throughout the training period. This finding underscores the potential benefits of gradually increasing CAF intake for better absorption and body tolerance during the intervention (10, 18). Nevertheless, these strategies appeared to be effective only for short-duration training. Caution is warranted when applying these results to long-term training durations, and additional studies are required to better understand the long-term implications of progressive and consistent CAF intake on strength performance.

The results of the present study indicated that a 4-week PJT intervention resulted in small to moderate ESs in the improvements of Ppeak and Pmean among volleyball players. Notably, the consumption of 6 mg/kg of CAF, or an increase in CAF intake from 3 to 6 mg/kg, led to greater adaptations compared to other interventions (moderate vs. small ES). The primary effects of PJT that contributed to the enhancements in Ppeak and Pmean can be attributed to increased motor unit recruitment and more forceful muscle contractions (5, 25, 30). Specifically, PJT improved the rate of force development and engaged fast-twitch muscle fibers, resulting in greater force production (4, 6) as evidenced by the Wingate anaerobic power test. These findings align with previous research that emphasized the beneficial impact of PJT on athletes’ power output (5, 35). Additionally, the findings of our study revealed that the administration of 6 mg/kg of CAF, or a gradual increase from 3 to 6 mg/kg, resulted in more pronounced adaptations that support the ergogenic properties of CAF in power production. In fact, the intake of higher doses of CAF (such as 6 mg/kg or the range of 3 to 6 mg/kg) stimulates the sarcoplasmic reticulum to release increased amounts of calcium, as demonstrated in an *in vitro* study (36), which enhances the recruitment of motor units (11). This leads to more forceful muscular contractions and adaptations (13), ultimately resulting in greater improvements in Ppeak and Pmean.

Moreover, CAF enhances the production of neurotransmitters that activate the brain, leading to heightened alertness and an improved mood (12). It also aids in neuromuscular recruitment and decreases the time needed to achieve maximum effort during PJT (17), resulting in beneficial adaptations in the power output of volleyball players. Our research, however, revealed that a progressive CAF dosage strategy is equally effective as a consistent dosage over the 4-week PJT, potentially providing the way for new supplementation approaches. Nonetheless, while our findings suggest potential for short-term adaptation, the long-term effectiveness of such dosing strategies remains to be confirmed, and further research is necessary to fully comprehend the long-term effects of both progressive and consistent CAF consumption on Wingate anaerobic power performance.

Collectively, our results indicate that a lower overall CAF consumption, achieved through a 4-week intervention employing a novel supplementation strategy (gradually increasing from 3 to 6 mg/kg), yields comparable enhancements in the physical performance of volleyball players relative to a consistent high-dose (i.e., 6 mg/kg) CAF regimen. It appears that incrementally increasing CAF dosages may attenuate the potential for receptor desensitization while promoting the upregulation of key physiological pathways, such as heightened the Na^+^/K^+^ and improved calcium-handling efficacy (10, 11). These adaptive responses may contribute to sustained performance advantages by mitigating receptor downregulation and preserving neuromuscular sensitivity to CAF (12). Although calcium release and Na^+^/K^+^ activity are crucial in elucidating the acute effects of caffeine, their integration within the context of dosing strategies remains a comparatively under-investigated area (16). Gradual CAF administration may serve to minimize receptor desensitization and facilitate the upregulation of adaptive mechanisms, including enhanced Na^+^/K^+^ efficiency and calcium handling capacity (10, 36). Therefore, future research should examine the role of tolerance theory, balancing processes of desensitization and upregulation, to optimize the ergogenic potential of CAF in the context of training adaptations.

This research has several methodological constraints that warrant consideration. The first limitation is the small sample size, which presents valuable opportunities for future investigations. However, the sample size was determined through G*power analysis (17) to ensure statistical adequacy. Additionally, it is essential to note that the results are exclusively applicable to male volleyball players, thereby limiting their applicability to female athletes and individuals engaged in different sports disciplines. Moreover, the lack of laboratory measures to assess physiological and neuromuscular adaptations restricts the ability to elucidate the metabolic and muscular changes, including anaerobic capacity and physical performance enhancements, that may have occurred in the athletes. Accordingly, further research is needed to explore the ergogenic effects of CAF ingestion at varying dosages on performance adaptations following PJT. Given these limitations, the findings should be considered preliminary, highlighting the importance of additional studies to validate or refute our results.

## 5 Conclusion

Our study suggests that the implementation of a short-term PJT may lead to improvements in the exercise performance of volleyball players during the pre-season. Additionally, administering 6 mg/kg of caffeine or gradually increasing the dosage from 3 to 6 mg/kg of caffeine could enhance these adaptations. Given these results, it is recommended to consider high doses of caffeine as an ergogenic aid to optimize performance adaptations in volleyball players. Furthermore, the strategy of progressively increasing caffeine dosage proves to be as effective as maintaining a consistent dosage throughout the 4-week PJT, offering a viable option for caffeine supplementation with reduced overall consumption while achieving similar training benefits.

## Data Availability

The raw data supporting the conclusions of this article will be made available by the authors, without undue reservation.
